# Translating Sustainable Development Goals into Veterinary Action: A Structured Priority-Setting Framework

**DOI:** 10.3390/vetsci13090912

**Published:** 2026-09-04

**Authors:** Harun Yonar, Furkan Çağrı Beşoluk, Aynur Yonar, Mehmet Emin Tekin, Kamil Beşoluk

**Affiliations:** 1 Department of Veterinary Biostatistics, Faculty of Veterinary Medicine, Selcuk University, Konya 42130, Türkiye; furkan.besoluk@selcuk.edu.tr (F.Ç.B.); mtekin@selcuk.edu.tr (M.E.T.); 2 Department of Statistics, Faculty of Science, Selcuk University, Konya 42130, Türkiye; aynursahin@selcuk.edu.tr; 3 Department of Anatomy, Faculty of Veterinary Medicine, Selcuk University, Konya 42130, Türkiye; kbesoluk@selcuk.edu.tr

**Keywords:** One Health, veterinary sustainability, expert judgment, multi-criteria decision making, AHP, TOPSIS

## Abstract

Veterinary institutions need practical methods to translate the Sustainable Development Goals (SDGs) into profession-specific priorities. This study developed a two-phase priority-setting framework linking systematic SDG screening with multi-criteria decision analysis. First, all 169 SDG targets were screened using four veterinary-relevant dimensions. The 48 retained targets were synthesized into 27 preliminary concepts, refined through two rounds of structured internal expert evaluation, and harmonized by the research team into 27 final decision alternatives. Second, Budgetary Burden, Accessibility and Scope, Implementation Timeline, and Sustainability Impact were weighted using the Analytic Hierarchy Process (AHP), and the 27 alternatives were ranked using TOPSIS. Robustness was assessed through Monte Carlo criterion-weight perturbation, score aggregation, and leave-one-expert-out analyses. Budgetary Burden (0.4025) and Sustainability Impact (0.3526) received the largest weights. Prevention of Zoonotic Diseases ranked first (C_i_ = 0.8272), followed by Awareness-Raising Campaigns, Animal Welfare Inspections on Commercial Animal Farms, and Audits of Food Safety and Production Process Compliance Standards. Criterion-weight perturbation and score aggregation largely preserved the highest-priority set, whereas leave-one-expert-out analysis showed greater sensitivity to panel composition. The framework should therefore be interpreted as a transparent, context-dependent, priority-setting tool rather than as an externally validated or universally applicable hierarchy.

## Simple Summary

Veterinary institutions can contribute to sustainable development in many ways, but broad global goals do not indicate which actions should be implemented first. We developed a transparent priority-setting approach that converts relevant global sustainability targets into 27 veterinary actions and compares them according to budgetary burden, accessibility and scope, implementation timeline, and expected sustainability impact. Within the present five-member internal expert panel, Prevention of Zoonotic Diseases received the highest relative implementation priority. The main contribution is therefore not a universal ranking of veterinary actions, but an auditable planning approach that institutions can adapt using broader stakeholder participation and local evidence on resource requirements, reach, implementation conditions, and expected sustainability outcomes.

## 1. Introduction

Sustainable development requires coordinated progress across environmental integrity, social well-being, economic resilience, and institutional capacity [1]. The United Nations 2030 Agenda operationalizes this objective through 17 Sustainable Development Goals (SDGs) and 169 targets [2]. Global progress remains uneven, reinforcing the need to translate broad sustainability commitments into sector-specific actions that can be prioritized under real-world institutional constraints [3]. Structured approaches to SDG interactions provide useful foundations for this translation problem [4,5,6].

Veterinary medicine is particularly relevant because its responsibilities extend across animal health and welfare, food safety, zoonotic-disease prevention, antimicrobial resistance, livestock production, environmental interfaces, and public health. These responsibilities place veterinarians within the human–animal–environment nexus emphasized by contemporary One Health frameworks [7,8,9,10]. Professional organizations have likewise linked veterinary practice to sustainable food systems and multiple SDGs [11,12,13].

The existing veterinary sustainability literature has established important components of this agenda but remains distributed across different domains. Research has addressed animal welfare in relation to the SDGs [14], environmental sustainability in clinical practice and teaching hospitals [15,16,17,18,19,20], climate and sustainability education [21,22,23], One Welfare [24], profession-level SDG mapping [25], and implementation of One Health strategies [26]. Country- and target-level SDG trajectory methods further illustrate the importance of moving from broad goals toward decision-relevant priorities [27]. Within the literature reviewed for the present study, however, we did not identify a single veterinary application linking systematic consideration of all 169 SDG targets to profession-specific action development and quantitative implementation prioritization.

Multi-criteria decision-making (MCDM) methods are useful when priorities depend on several potentially competing dimensions. AHP provides a structured approach to eliciting criterion weights, whereas TOPSIS ranks alternatives according to their relative proximity to an ideal solution [28,29]. Combined AHP–TOPSIS applications have been used in health, agriculture, and environmental decision problems in which feasibility, resource burden, and expected benefit must be considered jointly [30,31,32,33]. In the present study, these methods are used as transparent decision-support tools rather than as a claim of algorithmic novelty.

Accordingly, this study aimed to develop an auditable pathway linking systematic SDG screening to profession-specific action development and multi-criteria implementation prioritization. The contribution is integrative: target screening, structured internal expert refinement, researcher-led harmonization, AHP criterion weighting, TOPSIS ranking, and robustness assessment were connected within one traceable veterinary application. The resulting priorities are interpreted as conditional on the present panel and criterion structure rather than as an externally validated professional consensus.

## 2. Materials and Methods

### 2.1. Study Design

**Figure 1:**
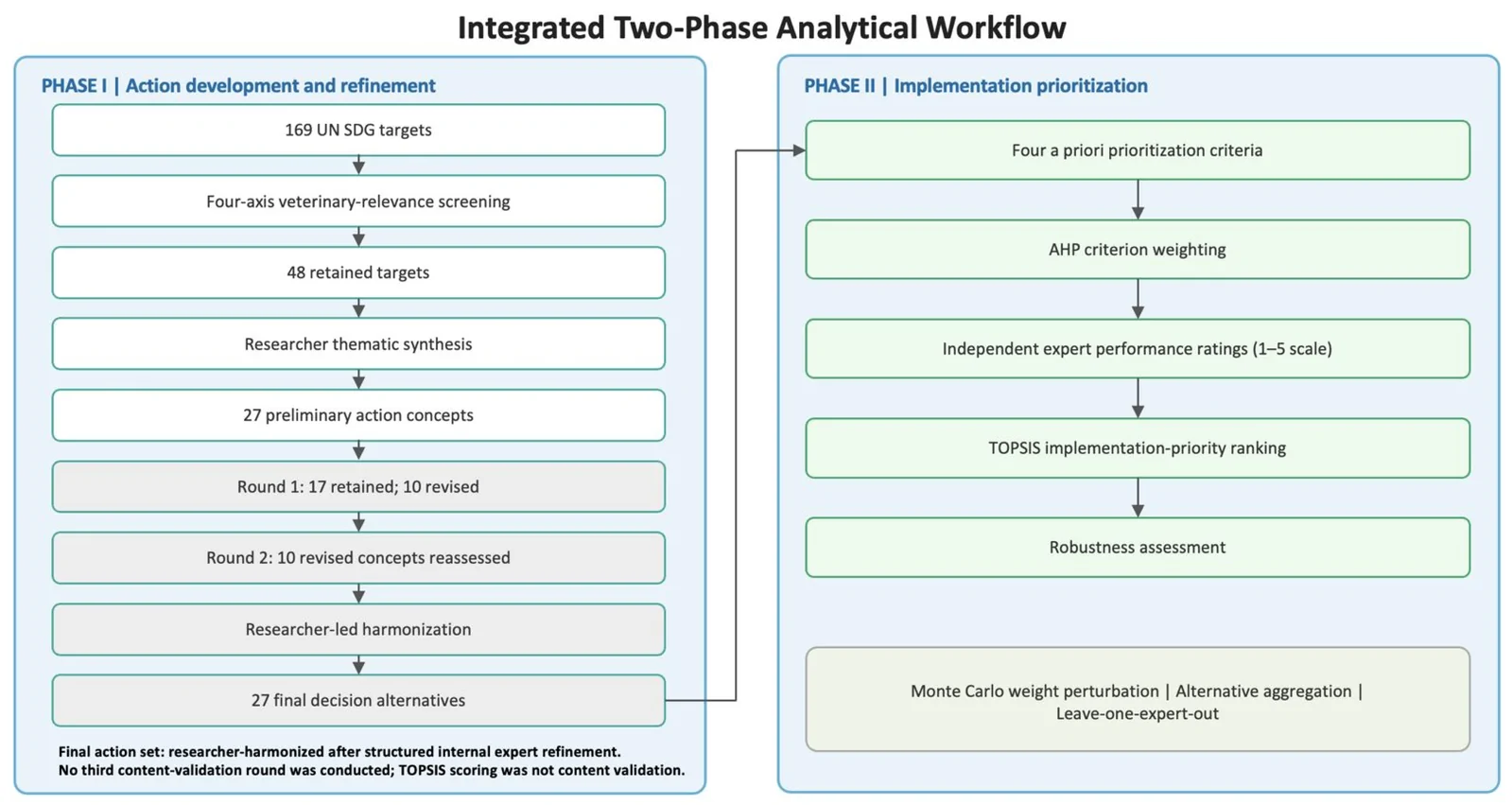
Integrated two-phase analytical workflow. The final action set was researcher-harmonized after structured internal expert refinement; TOPSIS performance scoring was not treated as content validation.

A two-phase decision-support design was used. Phase I screened all 169 SDG targets, synthesized retained targets into 27 preliminary veterinary sustainability concepts, refined those concepts through two rounds of structured internal expert evaluation, and then harmonized the expert-refined conceptual pool into 27 final decision alternatives. Phase II weighted four implementation-priority criteria using AHP and ranked the final alternatives using TOPSIS. Robustness was evaluated through weight perturbation, alternative score aggregation, and leave-one-expert-out analyses. The workflow is summarized in Figure 1; detailed stage-level audit records are provided in Supplementary File S1.

### 2.2. SDG Screening and Preliminary Action Development

A four-axis deductive coding framework was defined before full screening. It drew on structured SDG-interaction methods [4,5,6], One Health and veterinary SDG-mapping approaches [8,9,34], and deductive qualitative-content-analysis principles [35,36]. Two researchers independently coded each target for veterinary relevance, operational actionability, One Health/sustainability alignment, and professional role specificity. Retention required simultaneous satisfaction of the minimum eligibility condition on all four axes. Agreement was assessed before consensus using linearly weighted Cohen’s kappa, with exact agreement weighted 1, adjacent-category disagreement 0.5, and extreme-category disagreement 0 [37].Detailed category definitions and decision rules are provided in Supplementary Methods S1, and inter-rater reliability is summarized in Supplementary Table S1 of Supplementary File S1.

The 48 retained targets were jointly synthesized by the two researchers according to shared professional function, implementation mechanism, actionability, and expected sustainability outcome. The mapping was intentionally many-to-many rather than one-to-one, producing 27 preliminary candidate concepts.

### 2.3. Expert Panel and Structured Internal Refinement

A purposively assembled panel of five academics participated in the expert-evaluation phase. Four were affiliated with a Faculty of Veterinary Medicine and one with a Faculty of Science; three panel members specialized in veterinary biostatistics, one in basic sciences, and one in statistics. Academic and professional experience ranged from 5 to 40 years, with a median of 16 years. The panel was assembled to combine veterinary academic and quantitative decision-method expertise, not to provide statistical representation of the veterinary profession. Its disciplinary breadth was therefore limited for profession-wide sustainability assessment.

All five panel members were also members of the study team. Two had conducted the initial SDG screening and thematic synthesis; the other three had not participated in those initial stages. The full five-member panel began its role with independent evaluation of the 27 preliminary concepts and later completed the AHP and TOPSIS assessments. All refinement ratings, pairwise comparisons, and performance ratings were completed individually without access to other members’ responses or to a predetermined criterion ranking. This design maintained continuity across stages but reduced independence between framework development and evaluation; the process is therefore treated as structured internal expert elicitation rather than external validation.

Each preliminary concept was rated on veterinary relevance, SDG alignment, clarity, and operational actionability using a four-point scale (1 = not appropriate, 2 = major revision required, 3 = minor revision required, 4 = appropriate). Retention without major revision required ratings of 3 or 4 from at least four of five experts on every dimension and no unresolved major qualitative concern [38,39]. The 80% criterion was not an automatic deletion rule: concepts with remediable problems were revised and reassessed. After Round 1, 17 concepts were retained without major revision and 10 were revised; all 10 revised concepts received acceptable ratings from all five experts across all four dimensions in Round 2. Detailed round-level outcomes are presented in Supplementary Tables S2 and S3 of Supplementary File S1.

### 2.4. Researcher-Led Harmonization of the Final Action Set

After expert refinement, the research team harmonized the conceptual pool for use as MCDM decision alternatives. Harmonization included consolidation, splitting, reframing, and operationalization, so one-to-one equivalence in title and scope between preliminary and final actions was not preserved. Reciprocal consolidation and disaggregation operations left the total number of final alternatives at 27. This was a researcher-led structuring phase rather than a third consensus or content-validation round. The same panel subsequently rated the final alternatives against the TOPSIS performance criteria, but these ratings assessed implementation characteristics and did not validate conceptual content, wording, scope, or SDG alignment. The final action set is therefore described throughout as researcher-harmonized following structured internal expert refinement. Key consolidation and disaggregation operations are summarized in Supplementary Table S4 of Supplementary File S1, while the complete harmonization audit is retained in Supplementary File S1.

### 2.5. Prioritization Criteria and AHP–TOPSIS Procedure

Four criteria were defined a priori by the research team: Budgetary Burden, Accessibility and Scope, Implementation Timeline, and Sustainability Impact. The structure was deliberately parsimonious. Budgetary Burden, Accessibility and Scope, and Implementation Timeline represent complementary aspects of implementation feasibility, whereas Sustainability Impact represents expected multidimensional benefit. The four criteria were not derived statistically or selected through a separate expert-consensus round and are not presented as universal or exhaustive. Epidemiological urgency, regulatory feasibility, equity, stakeholder acceptability, institutional capacity, and long-term maintenance are among the dimensions that could reasonably be incorporated in context-specific adaptations. In particular, Implementation Timeline denotes expected rapidity of initiation/operationalization and should not be interpreted as epidemiological or political urgency. Full operational definitions and common 1–5 scoring anchors are provided in Supplementary Table S5 of Supplementary File S1.

For AHP, each expert completed the six unique pairwise comparisons among the four criteria using Saaty’s 1–9 scale [28]. Experts were given equal importance. Individual consistency was assessed using the consistency ratio (CR; RI = 0.90 for four criteria), with CR <= 0.10 considered acceptable. Corresponding pairwise judgments were aggregated using the element-wise geometric mean (aggregation of individual judgments), and group weights were derived using row geometric means [40]. Individual AHP judgments and CR values are reported in Supplementary Table S6 of Supplementary File S1.

For TOPSIS, all five experts received the same 27 final action titles together with the common criterion definitions, criterion directions, and criterion-specific 1–5 performance anchors. No separately archived action-specific operational-description sheet was used. All experts independently rated all actions. Because the ratings used a common 1–5 scale, the primary group decision matrix was formed by arithmetic averaging across experts. The matrix was vector-normalized and multiplied by the AHP weights; Budgetary Burden was treated as a cost criterion and the remaining criteria as benefits. Positive and negative ideal solutions, Euclidean distances, and closeness coefficients were then calculated following the standard TOPSIS procedure [29]. Larger closeness coefficients indicate greater relative implementation priority within the specified model, not greater intrinsic sustainability importance or empirical cost-effectiveness. The complete 27 × 4 aggregated decision matrix is provided in Supplementary Table S7 of Supplementary File S1.

### 2.6. Robustness, Concordance, and Software

Three robustness analyses were performed. First, each AHP weight was independently multiplied by a random factor drawn from Uniform (0.90, 1.10), renormalized to sum to 1, and the complete TOPSIS analysis was repeated for 1000 simulations using seed 12345; extended ±20% and ±30% perturbation scenarios were also examined [41]. Second, arithmetic aggregation of TOPSIS performance scores was compared with geometric aggregation. Third, five leave-one-expert-out scenarios simultaneously removed one expert from both AHP and TOPSIS inputs and recalculated the complete model. Rank stability was summarized using Kendall’s tau, rank shifts, top-set overlap, and top-ranked action identity; Kendall’s W was used descriptively for criterion-specific concordance [42]. Criterion-specific Kendall W values are reported in Supplementary Table S8 of Supplementary File S1.

All data processing and statistical analyses were performed using Python 3.11 and R version 4.5.0, with R-based work conducted in RStudio Desktop version 2025.05.1+513 [43,44]. English editing was performed using ChatGPT (GPT 5.5) (OpenAI, https://chatgpt.com).

## 3. Results

### 3.1. SDG Screening and Coding Reliability

Application of the pre-specified conjunctive rule retained 48 of 169 SDG targets (28.4%) for thematic synthesis and excluded 121 (71.6%). Before consensus, linearly weighted Cohen’s kappa coefficients were 0.952 for veterinary relevance, 0.931 for operational actionability, 0.874 for One Health/sustainability alignment, and 0.992 for professional role specificity; raw agreement rates were 95.9%, 94.1%, 89.3%, and 99.4%, respectively. Initial inclusion/exclusion decisions agreed for 166 of 169 targets (98.2%; Cohen’s kappa = 0.956), and three decision disagreements were resolved through consensus. These reliability results are summarized in Supplementary Table S1.

### 3.2. Expert Refinement and Final Researcher-Harmonized Action Set

The thematic synthesis produced 27 preliminary concepts. Seventeen were retained without major revision after Round 1, whereas 10 were revised and reassessed. All 10 revised concepts received acceptable ratings from all five experts on all four dimensions in Round 2. The round-level refinement record is summarized in Supplementary Tables S2 and S3. Researcher-led harmonization subsequently restructured the expert-refined conceptual pool through consolidation and disaggregation, yielding 27 final decision alternatives without preserving one-to-one title or scope equivalence with the preliminary pool (Supplementary Table S4). The final actions and their descriptive SDG associations are shown in Table 1.

### 3.3. AHP Criterion Weights and TOPSIS Ranking

All five individual AHP matrices satisfied CR <= 0.10 (range 0.0169–0.0355), and the aggregated group matrix had CR = 0.0226. Individual judgments and CR values are reported in Supplementary Table S6. Budgetary Burden received the largest group weight (0.4025), followed by Sustainability Impact (0.3526), Implementation Timeline (0.1420), and Accessibility and Scope (0.1029) (Table 2). Budgetary Burden and Sustainability Impact together accounted for 75.51% of total criterion weight.

Prevention of Zoonotic Diseases ranked first (Cᵢ = 0.8272), followed by Awareness-Raising Campaigns (0.7366), Animal Welfare Inspections on Commercial Animal Farms (0.7344), Audits of Food Safety and Production Process Compliance Standards (0.7298), and Education and Awareness (0.6771). The complete ranking is presented in Table 3; the underlying aggregated decision matrix is provided in Supplementary Table S7.

### 3.4. Robustness and Panel-Composition Sensitivity

The highest-priority core was stable to the tested criterion-weight perturbations and aggregation operator. In the ±10% Monte Carlo scenario, A19 remained first in all 1000 simulations; the mean Kendall tau versus baseline was 0.9852. A19 also remained first in all ±20% and ±30% simulations. Geometric rather than arithmetic aggregation of expert TOPSIS ratings produced Spearman rho = 0.9908 and Kendall tau = 0.9544, with identical top five and top ten action sets. Detailed Monte Carlo and aggregation-operator results are reported in Supplementary Table S9. Leave-one-expert-out results were more variable: Kendall tau ranged from 0.6866 to 0.8575, maximum absolute rank shifts ranged from 5 to 10, and A19 remained first in four of five scenarios; excluding E4 produced A26 as the top action. Full leave-one-expert-out results are provided in Supplementary Table S10. The main robustness findings are summarized in Table 4.

## 4. Discussion

### 4.1. Principal Findings and Contribution

This study links systematic SDG screening to veterinary action development and quantitative implementation prioritization within one auditable workflow. Its main contribution is therefore integrative rather than algorithmic. The framework makes explicit how broad global targets can be screened, translated into profession-specific concepts, internally refined, researcher-harmonized into decision alternatives, weighted, ranked, and stress-tested. This distinction is important because the final ranking is a planning output under a defined panel and criterion structure, not a claim of profession-wide consensus or external content validation.

The first phase narrowed 169 global targets to 48 targets meeting all four pre-specified veterinary-relevant conditions and then synthesized them into a manageable preliminary action pool. High pre-consensus agreement supports reproducibility of the screening rule itself. The expert stage, however, was deliberately an internal refinement mechanism: 10 concepts required revision, and the rule allowed remediable concepts to be revised rather than mechanically deleted. Researcher-led consolidation and disaggregation then improved the granularity of the final decision alternatives while changing the validation status of their final wording and scope.

### 4.2. Decision Structure and Interpretation of the Highest-Ranked Actions

Budgetary Burden and Sustainability Impact together accounted for 75.51% of the group AHP weight. For this panel, implementation priority therefore reflected mainly the balance between resource burden and expected multidimensional benefit, while access and speed carried smaller weights. These weights should not be interpreted as a universal veterinary value structure; a model that explicitly adds urgency, equity, regulatory feasibility, institutional capacity, or stakeholder acceptability could yield a different hierarchy.

A19 ranked first because it combined a comparatively low Budgetary Burden score (2.0) with high Sustainability Impact (4.2) and relatively high Accessibility and Scope (3.6), producing a favorable balance across the two most heavily weighted criteria. A24 had an even lower budget score (1.8) but a lower Sustainability Impact score (3.4), illustrating that ranking was not driven by budget alone. The prominence of zoonotic-disease prevention is substantively compatible with the veterinarian’s role at the human–animal–environment interface and with multisectoral One Health guidance [7,9,45], but that compatibility should not be interpreted as independent validation of the ranking.

### 4.3. Relation to the Veterinary Sustainability and MCDM Literature

Veterinary sustainability research has emphasized environmental practice, institutional implementation, staff capacity, education, and stakeholder engagement [15,16,17,18,19,20,21,22,23,46]. Kern-Allely et al. [20], for example, used a two-round expert process to develop clinical environmental sustainability actions and considered implementation effort alongside impact. The present framework is broader in substantive scope, extending across zoonoses, livestock and food systems, service access, education, community well-being, and ecosystem management; however, its small internal panel is substantially narrower than the stakeholder breadth desirable for profession-wide external validation.

The use of AHP and TOPSIS is consistent with applications in health, agriculture, and environmental management where multiple implementation dimensions must be balanced [30,31,32,33]. The robustness results qualify interpretation of the hierarchy: the high-priority core was stable to the tested criterion-weight perturbations and score-aggregation operator, whereas leave-one-expert-out analysis showed materially greater variability. The most important uncertainty in the present application therefore appears to arise from who is represented in the small panel rather than from the tested computational specifications.

### 4.4. Policy and Practice Implications

The framework is best viewed as a structured starting point for local priority setting rather than as a prescriptive implementation sequence. Under the present panel and four-criterion structure, zoonotic-disease prevention, awareness-raising, animal-welfare inspection, food-safety compliance auditing, and education and awareness formed the highest-ranked group. These actions may warrant further local assessment when institutions identify candidate priorities, but the present ranking alone does not establish implementation effectiveness, cost-effectiveness, or transferability across veterinary systems, including resource-constrained settings.

Before operational use, institutions should reassess criterion definitions and weights with stakeholders relevant to their setting, consider additional dimensions such as epidemiological urgency, equity, regulatory feasibility, and institutional capacity, and replace judgment-based performance scores with local cost, access, duration, and outcome data where available. In this way, the model functions as a transparent decision scaffold that can be adapted rather than a fixed universal ranking.

### 4.5. Limitations and Future Research

Several limitations define the appropriate interpretation of this study. First, the five-member panel was small, internal, and disciplinarily concentrated. Although it combined veterinary academic and quantitative expertise, it did not directly represent veterinary public health/One Health, animal welfare, epidemiology, food safety, livestock production, clinical practice, or environmental sustainability as independent external specialties. All five experts were members of the study team, and two had participated in the earlier screening and synthesis stages. Independent completion of ratings limited direct interpersonal influence but did not remove possible anchoring, confirmation bias, or shared conceptual assumptions. The leave-one-expert-out findings empirically demonstrate the influence of panel composition (Supplementary Table S10).

Second, all 27 preliminary concepts remained in the framework because the refinement rule was revision-oriented rather than a mechanical exclusion rule; this should not be interpreted as evidence that an independent profession-wide panel considered every initial concept valid. Third, the final 27 alternatives were researcher-harmonized after expert refinement. Consolidation and disaggregation altered titles and scopes, and no third content-validation round was conducted. TOPSIS performance scoring cannot substitute for content validation; independent external evaluation of the final wording, scope, and SDG alignment is therefore an important next step.

Fourth, experts rated common title-level alternatives using standardized criterion definitions and scoring anchors, but no separately archived action-specific operational-description sheet was used. Broad titles may therefore have been interpreted differently during scoring, potentially contributing to the low criterion-specific concordance reported in Supplementary Table S8. The present data cannot retrospectively distinguish interpretation variability from genuine differences in expert judgment. Future applications should provide standardized action-specific operational descriptions or vignettes before performance scoring.

Finally, the four criteria were defined a priori and do not exhaust the dimensions relevant to implementation. Scores represent expert judgments rather than measured costs, timelines, access, or outcomes, and TOPSIS treats the common 1–5 ratings as approximately interval-scaled for aggregation and distance calculations even though they originate from an ordinal response scale. AHP and TOPSIS also depend on scale use, criterion independence, normalization, compensability, and distance assumptions. The robustness analyses addressed criterion-weight perturbation, aggregation operator, and panel composition, but not the full range of model uncertainty. Future studies should externally validate the final action set with larger and more heterogeneous panels, compare alternative criterion structures and MCDM models, incorporate empirical implementation data, and evaluate prioritized actions prospectively across institutions or countries.

## 5. Conclusions

This study contributes a transparent pathway for moving from global sustainability targets to profession-specific veterinary implementation priorities. Its principal contribution is the integration of SDG screening, structured internal expert refinement, researcher-led harmonization, AHP weighting, TOPSIS ranking, and robustness assessment within one traceable decision-support process. Under the present five-member panel and four-criterion structure, Budgetary Burden and Sustainability Impact had the greatest influence on priority setting, and Prevention of Zoonotic Diseases received the highest relative implementation priority.

The resulting hierarchy should be interpreted as a context-dependent planning output rather than as an externally validated professional consensus or an empirical cost-effectiveness ranking. Its practical value lies in providing a reproducible structure that veterinary institutions can re-weight, externally validate, and update using broader stakeholder participation and local cost, feasibility, and outcome data.

## Figures and Tables

**Table 1:** Researcher-harmonized final veterinary sustainability actions and descriptive SDG associations.

No.	Final Action	Functional Domain	Associated SDGs
1	Waste Management	Environmental and Ecosystem Management	12, 13, 15
2	Energy Efficiency	Environmental and Ecosystem Management	7, 13
3	Water Conservation	Environmental and Ecosystem Management	6, 12, 15
4	Use of Environmentally Friendly Products and Agricultural Practices	Environmental and Ecosystem Management	12, 13, 15
5	Maintaining Ecological Balance	Environmental and Ecosystem Management	13, 14, 15
6	Protecting Biodiversity and Habitat Restoration	Environmental and Ecosystem Management	15, 13
7	Reducing the Environmental Impact of Veterinary Drugs and Promoting Responsible Drug Use	Environmental and Ecosystem Management	12, 3, 15
8	Vaccinations and Health Screening Programs	Preventive Health and One Health	3
9	More Efficient and High-Quality Production	Sustainable Livestock and Food Systems	8, 9, 12
10	Ensuring the Economic Sustainability of Livestock Enterprises	Sustainable Livestock and Food Systems	8, 12
11	Increasing the Productivity of Livestock Enterprises	Sustainable Livestock and Food Systems	2, 8, 12
12	Investing in Veterinary Clinics in Rural Areas	Service Access, Infrastructure, and Digitalization	3, 9, 10
13	Contributing to the Local Economy and Sustainable Production	Sustainable Livestock and Food Systems	8, 12
14	Community Services	Community Well-Being and Social Responsibility	3, 11, 17
15	Certified Organic Products	Sustainable Livestock and Food Systems	12, 3
16	Telemedicine and Digital Tools	Service Access, Infrastructure, and Digitalization	3, 9, 10
17	Research and Development	Education, Innovation, Communication, and Professional Development	9, 4, 17
18	Human–Animal Interaction	Community Well-Being and Social Responsibility	3, 15
19	Prevention of Zoonotic Diseases	Preventive Health and One Health	3, 15
20	Continuing Professional Development and Practical Training	Education, Innovation, Communication, and Professional Development	4, 8
21	Education and Awareness	Education, Innovation, Communication, and Professional Development	4, 3
22	Animal Welfare Inspections on Commercial Animal Farms	Sustainable Livestock and Food Systems	2, 12, 15
23	Accessibility in Veterinary Clinics	Service Access, Infrastructure, and Digitalization	3, 10
24	Awareness-Raising Campaigns	Education, Innovation, Communication, and Professional Development	4, 12
25	Social Responsibility Projects	Community Well-Being and Social Responsibility	3, 4, 10, 17
26	Audits of Food Safety and Production Process Compliance Standards	Sustainable Livestock and Food Systems	2, 3, 12
27	Veterinary and Community Relations	Education, Innovation, Communication, and Professional Development	3, 11, 17

**Table 2:** Group AHP criterion weights.

Criterion	Weight	Rank
Budgetary Burden	0.4025	1
Sustainability Impact	0.3526	2
Implementation Timeline	0.1420	3
Accessibility and Scope	0.1029	4

**Table 3:** AHP–TOPSIS implementation-priority ranking of the 27 final actions.

Rank	Action No.	Action	Cᵢ
1	19	Prevention of Zoonotic Diseases	0.8272
2	24	Awareness-Raising Campaigns	0.7366
3	22	Animal Welfare Inspections on Commercial Animal Farms	0.7344
4	26	Audits of Food Safety and Production Process Compliance Standards	0.7298
5	21	Education and Awareness	0.6771
6	1	Waste Management	0.6706
7	18	Human–Animal Interaction	0.6404
8	20	Continuing Professional Development and Practical Training	0.6307
9	7	Reducing the Environmental Impact of Veterinary Drugs and Promoting Responsible Drug Use	0.6000
10	25	Social Responsibility Projects	0.5817
11	17	Research and Development	0.5754
12	15	Certified Organic Products	0.5470
13	10	Ensuring the Economic Sustainability of Livestock Enterprises	0.5424
14	12	Investing in Veterinary Clinics in Rural Areas	0.5325
15	5	Maintaining Ecological Balance	0.4962
16	13	Contributing to the Local Economy and Sustainable Production	0.4718
17	6	Protecting Biodiversity and Habitat Restoration	0.4344
18	8	Vaccinations and Health Screening Programs	0.4262
19	27	Veterinary and Community Relations	0.4015
20	9	More Efficient and High-Quality Production	0.3532
21	16	Telemedicine and Digital Tools	0.3266
22	14	Community Services	0.3043
23	4	Use of Environmentally Friendly Products and Agricultural Practices	0.2650
24	2	Energy Efficiency	0.2441
25	11	Increasing the Productivity of Livestock Enterprises	0.2420
26	3	Water Conservation	0.2256
27	23	Accessibility in Veterinary Clinics	0.1483

**Table 4:** Summary of principal robustness analyses.

Analysis	Principal Finding	Interpretation
Monte Carlo ±10% (1000 runs; seed 12345)	A19 ranked first in 100% of runs; mean Kendall tau = 0.9852.	High stability to local criterion-weight uncertainty.
Extended ±20% and ±30%	A19 ranked first in 100% of runs in both scenarios.	Top action stable under broader tested weight perturbations.
Arithmetic vs. geometric TOPSIS score aggregation	Spearman rho = 0.9908; Kendall tau = 0.9544; Top 5 and Top 10 sets identical.	Little dependence on the tested aggregation operator.
Leave-one-expert-out	Kendall tau = 0.6866–0.8575; A19 first in 4/5 scenarios; A26 first when E4 was excluded.	Greater sensitivity to panel composition than to tested analytical specifications.

## Data Availability

The data presented in this study are available on request from the corresponding author. The data are not publicly available due to privacy and ethical restrictions, as the underlying individual-level expert judgments may be identifiable because of the small and named expert panel.

## Supplementary Materials

The following supporting information can be downloaded at: https://www.mdpi.com/article/10.3390/vetsci13090912/s1, Supplementary File S1: Detailed_Methods_and_Robustness.docx (Supplementary Tables S1–S10).
