# Supplementary material for: Translating Sustainable Development Goals into Veterinary Action: A Structured Priority-Setting Framework
Source: Vet Sci. 2026 Sep 4;13(9):912. doi: 10.3390/vetsci13090912 (PMC13611875; doi:10.3390/vetsci13090912)
Supplement: Supplementary file 1 [file vetsci-13-00912-s001.zip › vetsci-4513531-supplementary.pdf]

Supplementary Materials

Development and Prioritization of Veterinary Sustainability Actions: An Integrated SDG Screening and AHP–TOPSIS Framework

This document contains detailed methodological and analytical material moved from the main manuscript during revision. The complete audit workbook is supplied separately as Supplementary File S1. The final actions remain researcher-harmonized following structured internal expert refinement; this file does not introduce a new validation round.

Supplementary Methods S1. Detailed SDG Screening Framework

Axis	Categories	Retention-eligible categories	Operational focus
Veterinary relevance	Direct / Indirect / Unrelated	Direct or Indirect	Meaningful overlap with veterinary responsibilities, animal health/welfare, veterinary public health, food safety, or livestock production.
Operational actionability	Yes / Partial / No	Yes or Partial	Ability to translate the target into a field-applicable professional action, advisory role, protocol, surveillance activity, education activity, or policy contribution.
One Health/sustainability alignment	Strong / Moderate / None	Strong or Moderate	Meaningful human-animal-environment or sustainability connection.
Professional role specificity	Explicit / Partial / Unclear	Explicit or Partial	Identifiable veterinary role, either direct or through interdisciplinary collaboration.

Conjunctive rule: a target was retained only if it satisfied the minimum eligibility category on all four axes. Linearly weighted Cohen's kappa used weights of 1 for exact agreement, 0.5 for adjacent-category disagreement, and 0 for extreme-category disagreement.

Supplementary Table S1. Inter-rater reliability for the four SDG screening axes.

Axis	Agreements	Disagreements	Raw agreement	Weighted kappa
Veterinary relevance	162	7	95.9%	0.952
Operational actionability	159	10	94.1%	0.931
One Health/sustainability alignment	151	18	89.3%	0.874
Professional role specificity	168	1	99.4%	0.992

Initial inclusion/exclusion agreement was 166/169 targets (98.2%; unweighted Cohen's kappa = 0.956). Three provisional decision disagreements were resolved through consensus after independent coding.

Supplementary Methods S2. Structured Internal Expert Refinement

Acceptable support was defined as a score of 3 or 4. Retention without major revision required acceptable support from at least 4/5 experts on every dimension and no unresolved major qualitative concern. The threshold was not an automatic deletion rule; remediable concepts could be revised and reassessed.

Supplementary Table S2. Round-1 and Round-2 refinement outcomes.

Stage	Outcome	Interpretation
Round 1	17 retained without major revision	Met the retention-without-major-revision rule.
Round 1	10 revised and forwarded to Round 2	Revision addressed clarity, scope, SDG alignment, overlap, veterinary specificity, or actionability.
Round 1	0 permanently removed	The process was revision-oriented rather than a mechanical deletion procedure.
Round 2	10/10 revised concepts retained	All five experts assigned acceptable ratings on all four dimensions.
End of expert refinement	27 expert-refined preliminary concepts	Forwarded to researcher-led harmonization; not equivalent to final wording/scope.

Supplementary Table S3. Ten revised preliminary concepts reassessed in Round 2.

Code	Revised concept title	Round-2 outcome
P3	Economic Sustainability and Profitability of Livestock Enterprises	100% acceptable support on all four dimensions
P5	Veterinary Field Advisory and Farmer Training for Sustainable Livestock Management	100% acceptable support on all four dimensions
P8	Expanding Veterinary Service Reach through Mobile and Community-Based Models	100% acceptable support on all four dimensions
P15	Slaughter Facility and Animal Product Processing Compliance Audits	100% acceptable support on all four dimensions
P16	Veterinary Advisory on Livestock-Related Nutrient and Pollution Management	100% acceptable support on all four dimensions
P17	Wildlife Disease Surveillance in Wetland and Aquatic Habitats	100% acceptable support on all four dimensions
P18	On-Farm Biogas, Waste-to-Energy and Carbon Footprint Advisory in Livestock Operations	100% acceptable support on all four dimensions
P23	Endangered Species Protection and Invasive Animal Species Control	100% acceptable support on all four dimensions
P26	Veterinary Innovation: AI-Assisted Diagnostics, Data Analytics and Research Development	100% acceptable support on all four dimensions
P27	Veterinary Professional Engagement in Multi-Stakeholder Sustainability Platforms	100% acceptable support on all four dimensions

Supplementary Methods S3. Researcher-Led Harmonization

The full audit bridge is provided in Supplementary File S1, worksheet S5_Harmonisation_Audit. Harmonization included consolidation, splitting, reframing, and operationalization. These operations changed title/scope equivalence; no third content-validation round was conducted.

Supplementary Table S4. Key consolidation/disaggregation examples from the harmonization audit.

Source preliminary concept(s)	Operation	Final action(s)
P1, P14, P15	Consolidated / operationalized	A26 - Audits of Food Safety and Production Process Compliance Standards
P2	Split	A9 - More Efficient and High-Quality Production; A11 - Increasing the Productivity of Livestock Enterprises
P9	Split / reframed	A1 - Waste Management; A7 - Reducing the Environmental Impact of Veterinary Drugs and Promoting Responsible Drug Use
P13	Split / operationalized	A21 - Education and Awareness; A24 - Awareness-Raising Campaigns
P27	Split / operationalized	A14 - Community Services; A18 - Human-Animal Interaction; A25 - Social Responsibility Projects; A27 - Veterinary and Community Relations

Supplementary Methods S4. Prioritization Criteria and Scoring Anchors

Supplementary Table S5. TOPSIS criterion definitions and common 1–5 anchors.

Criterion	Definition	Type	1 anchor	5 anchor
Budgetary Burden	Financial resources, personnel, equipment, and operating expenditure required; higher values indicate greater burden.	Cost	Very low burden	Very high burden
Accessibility and Scope	Extent to which the target population can access and benefit across geographic, digital, institutional, and social barriers.	Benefit	Very low	Very high
Implementation Timeline	Expected rapidity with which the action can be initiated and operationalized under existing or reasonably attainable conditions.	Benefit	Very slow / long-term	Very rapid / short-term
Sustainability Impact	Expected environmental, social, economic, animal-health, public-health, and professional contribution.	Benefit	Very low	Very high

Supplementary Methods S5. AHP Inputs and Group Aggregation

Supplementary Table S6. Individual AHP pairwise judgments and consistency ratios.

Expert	B/A	B/T	B/I	A/T	A/I	T/I	CR
E1	5	3	1	1	0.25	0.5	0.0222
E2	5	2	1	1	0.25	0.5	0.0320
E3	6	3	1	1	0.2	0.5	0.0355
E4	5	3	1	1	0.33	0.5	0.0198
E5	4	2	1	1	0.33	0.5	0.0169

B = Budgetary Burden; A = Accessibility and Scope; T = Implementation Timeline; I = Sustainability Impact. Group weights: Budgetary Burden 0.4025; Accessibility and Scope 0.1029; Implementation Timeline 0.1420; Sustainability Impact 0.3526. Group CR = 0.0226.

Supplementary Methods S6. Aggregated TOPSIS Decision Matrix

Supplementary Table S7. Arithmetic-mean 27 × 4 group TOPSIS decision matrix.

Action	Budgetary Burden	Accessibility and Scope	Implementation Timeline	Sustainability Impact
A1	2.6	3.0	2.8	4.6
A2	3.6	3.2	3.2	3.4
A3	4.2	3.4	3.0	4.2
A4	3.6	3.6	3.2	3.6
A5	3.0	3.4	3.6	3.8
A6	3.2	3.4	3.0	4.0
A7	2.8	3.6	3.4	4.2
A8	3.2	3.8	3.4	3.8
A9	3.6	3.2	3.8	4.2
A10	2.8	3.8	3.4	3.6
A11	3.8	3.4	4.0	3.6
A12	3.0	3.8	3.4	4.2
A13	3.0	3.2	3.4	3.6
A14	3.4	3.4	3.2	3.2
A15	3.0	3.4	3.4	4.4
A16	3.4	4.0	2.6	3.6
A17	2.8	3.4	3.2	4.0
A18	2.4	3.8	3.6	3.4
A19	2.0	3.6	3.0	4.2
A20	2.6	3.4	2.8	4.0
A21	2.4	3.6	3.0	3.8
A22	2.2	3.4	2.6	4.0
A23	4.0	3.4	3.2	3.6
A24	1.8	3.4	3.4	3.4
A25	2.6	3.6	3.4	3.4
A26	2.2	3.8	3.0	3.8
A27	3.2	3.6	3.2	3.6

Supplementary Methods S7. Robustness and Concordance

Supplementary Table S8. Criterion-specific Kendall's W concordance coefficients for TOPSIS ratings.

Criterion	Kendall's W
Budgetary Burden	0.312
Accessibility and Scope	0.079
Implementation Timeline	0.172
Sustainability Impact	0.193

Supplementary Table S9. Monte Carlo and aggregation-operator robustness summary.

Analysis	Key result
+/-10% Monte Carlo, 1,000 runs, seed 12345	A19 first in all runs; mean Kendall tau = 0.9852. A24 mean rank 2.555 (SD 0.725; 2–4), A22 2.705 (0.671; 2–4), A26 3.740 (0.439; 3–4).
+/-20% Monte Carlo, 1,000 runs, seed 12345	Mean Kendall tau = 0.9711; mean Top-5 overlap = 4.625/5; mean Top-10 overlap = 9.625/10; A19 first in all runs.
+/-30% Monte Carlo, 1,000 runs, seed 12345	Mean Kendall tau = 0.9574; mean Top-5 overlap = 4.590/5; mean Top-10 overlap = 9.532/10; A19 first in all runs.
Arithmetic vs geometric aggregation	Spearman rho = 0.9908; Kendall tau = 0.9544; Top-5 overlap = 5/5; Top-10 overlap = 10/10; maximum absolute rank shift = 3.

Supplementary Table S10. Leave-one-expert-out panel-composition sensitivity.

Excluded expert	Kendall tau	Max shift	Top-ranked action	Top-5 overlap	Top-10 overlap	Reduced-panel CR
E1	0.8348	5	A19 - Prevention of Zoonotic Diseases	3/5	9/10	0.0231
E2	0.8575	5	A19 - Prevention of Zoonotic Diseases	4/5	10/10	0.0212
E3	0.8006	7	A19 - Prevention of Zoonotic Diseases	4/5	8/10	0.0203
E4	0.6866	10	A26 - Audits of Food Safety and Production Process Compliance Standards	4/5	6/10	0.0244
E5	0.8120	6	A19 - Prevention of Zoonotic Diseases	4/5	9/10	0.0248

Supplementary Methods S8. Reproducibility and Code Provenance

The final master workbook preserves the primary raw inputs and analytical outputs. The archived 01-Aug-2026 R script AHP_TOPSIS_Expert_Bootstrap_Robustness.R was prepared as part of the verification/robustness workflow. It reconstructs the AHP group weights, verifies the arithmetic-mean TOPSIS decision matrix and baseline closeness coefficients against the reported results, and then implements an expert-level bootstrap conditional on the observed five-expert panel. Supplementary Data S1 (current_TOPSIS_raw_scores.xlsx) was exported directly from worksheet S8_Raw_Scores of the final reviewer-backup workbook and converted only from wide format to long format; no rating values were altered. Historical custom Python source files referred to in the submitted manuscript are not present in the retained 01-Aug-2026 archive and have therefore not been reconstructed or represented as original code.
